# Experimental and Theoretical Study of the Kinetics of the CH_3_ + HBr → CH_4_ + Br Reaction and the Temperature Dependence of the Activation Energy of CH_4_ + Br → CH_3_ + HBr

**DOI:** 10.1021/acs.jpca.3c03685

**Published:** 2023-08-10

**Authors:** Yuri Bedjanian, Péter Szabó, György Lendvay

**Affiliations:** † Institut de Combustion, Aérothermique, Réactivité et Environnement (ICARE), CNRS, Orléans Cedex 2 45071, France; ‡ Department of Chemistry, KU Leuven, Celestijnenlaan, 200F, Leuven 3001, Belgium; § Royal Belgian Institute for Space Aeronomy (BIRA-IASB), Avenue Circulaire 3, Brussels 1180, Belgium; ∥ Institute of Materials and Environmental Chemistry, Research Centre for Natural Sciences, Magyar tudósok krt. 2., Budapest H-1117, Hungary; ⊥ Center for Natural Sciences, Faculty of Engineering, University of Pannonia, Egyetem u. 10., Veszprém 8200, Hungary

## Abstract

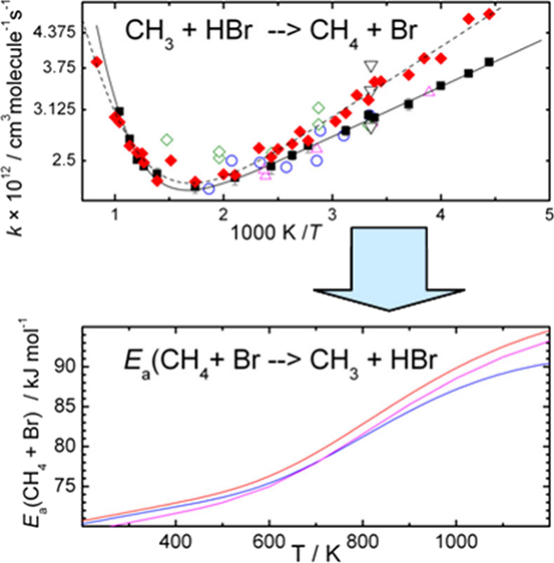

The rate coefficient of the reaction of CH_3_ with HBr was measured and calculated in the temperature range 225–960 K. The results of the measurements performed in a flow apparatus with mass spectrometric detection agree very well with the quasiclassical trajectory calculations performed on a previously developed potential energy surface. The experimental rate coefficients are described well with a double-exponential fit, *k*_1_(exp) = [1.44 × 10^–12^ exp(219/*T*) + 6.18 × 10^–11^ exp(−3730/*T*)] cm^3^ molecule^–1^ s^–1^. The individual rate coefficients below 500 K accord with the available experimental data as does the slightly negative activation energy in this temperature range, −1.82 kJ/mol. At higher temperatures, the activation energy was found to switch sign and it rises up to about an order of magnitude larger positive value than that below 500 K, and the rate coefficient is about 50% larger at 960 K than that around room temperature. The rate coefficients calculated with the quasiclassical trajectory method display the same tendencies and are within about 8% of the experimental data between 960 and 300 K and within 25% below that temperature. The significant variation of the magnitude of the activation energy can be reconciled with the tabulated heats of formation only if the activation energy of the reverse CH_4_ + Br reaction also significantly increases with the temperature.

## Introduction

One of the most reliable sources of the enthalpies of formation of chemical compounds is calorimetry, most commonly, measurement of their heats of combustion.^1^ The method cannot be used for unstable species, such as free radicals that cannot be placed in pure form into a calorimeter. For such species, reaction kinetics provides a way to determine their enthalpies of formation, based on the fact that in thermal equilibrium, the rates of the forward and reverse reactions are equal so that at a given temperature, the reaction enthalpy equals the difference of the forward and reverse activation energies.^2,3^ The reaction enthalpy, in turn, is the difference of the enthalpies of formation of the products and the reactants, and if all but one of these quantities are known, the missing one can be calculated from the reaction enthalpy. According to the second-law method, the reaction enthalpy is determined directly from the activation energies.^2,4^ Another option is the third-law method,^3,4^ in which the equilibrium constant is determined from the rate coefficients for the forward and reverse rate coefficients at a given temperature, and the reaction enthalpy is derived from the Gibbs free energy change of the reaction obtained from the van’t Hoff relation and the entropy change calculated using statistical mechanical formulas.

The kinetic method has been extensively used for the determination of the enthalpies of formation of alkyl radicals. Among several classes of reactions,^5,6^ measurements of the rate coefficients of their reactions with HBr:
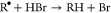
R0and the reverse reactions:
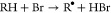
R-0were instrumental in deriving accurate thermochemical properties for alkyl radicals. At the end of the 1970s, the enthalpies of formation of alkyl radicals determined by the kinetic method using bromination and iodination reactions^6,7^ were systematically too low by 8–16 kJ/mol compared with those obtained from other sources, such as dissociation and the reverse radical recombination,^5,8−10^ as well as the body of heats of formation known in physical organic chemistry.^11,12^ The resolution of the discrepancy started with the experiments by Gutman and co-workers in 1988,^13,14^ in which the activation energy of R1-type reactions with C_1_–C_4_ alkyl radicals was found to be negative. The observation was soon confirmed by Nicovich *et al.*,^15^ Seakins *et al*.,^16^ and Seetula.^17^ The rates of the reverse reactions were derived from direct measurements of the hydrogen abstraction reaction from i-butane by Br atoms and earlier relative rate experiments. The negative activation energy was not consistent with what was known at the time about hydrogen abstraction reactions^6^ until ab initio calculations supported the mechanism proposed by Gutman and co-workers, viz., halogen hydrides form hydrogen-bonded van der Waals complexes with alkyl radicals that can decompose back to reactants or form products.^18^ The existence of such complexes has been proved in numerous ab initio calculations.^19−24^ Early theoretical studies^25^ utilizing RRKM theory applied to complex-forming bimolecular reactions^26^ confirmed the possibility that the activation energy can be negative at low temperatures. The attraction between the reactants is a key feature determining the kinetics and dynamics of reaction R1.

Detailed information is available on the potential energy surface of the prototype of alkyl + HBr type reactions, the reaction of methyl radicals with HBr,
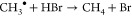
R1and the reverse
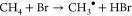
R-1

In this reaction, the depth of the van der Waals well is 10.38 kJ/mol, the top of the barrier to reaction is 7.61 kJ/mol below the reactant level, the reaction energy is −94.1 kJ/mol, and there is a product van der Waals well at 95.1 kJ/mol below the reactant level. (When the vibrational zero-point energies are included, the respective numbers are 3.39, 0.92, 71.42, and 72.51 kJ/mol, see Figure 1 in ref (27). The quoted numbers refer to the analytical potential energy surface that is based on the high-accuracy energy values derived by Czakó^23^ characterized by an estimated error of 0.6 kJ/mol.) The reaction dynamical studies^27−29^ performed using the quasiclassical trajectory (QCT) method (validated by reduced-dimensionality quantum scattering calculations^27^) indicate that the long-range attraction induces a capture-type behavior: At low collision energies, the excitation function (the reaction cross section as a function of collision energy) diverges as the collision energy is reduced. This kind of excitation function is associated with rate coefficients that, at low temperatures, increase as the temperature decreases, which is in agreement with the experimental observations. However, the character of the excitation function changes as the collision energy increases: the reaction cross sections pass a minimum and start rising again. The consequence is that the rate coefficients also increase when the temperature is increased. The existing experimental results agree with negative activation energy predicted by the QCT calculations for the low-temperature region. One can notice, however, that the rate coefficients at the highest temperatures in the experiments by Seetula^17^ do not decrease further when the temperature increases, and one can surmise that they can even increase at higher temperatures. Theoretical modeling based on transition state theory^20,25,26^ and QCT calculations^28^ predicted a switch of the activation energy to positive values, but no experiments were performed above 500 K.

The purpose of the present paper is a combined experimental and theoretical study of the temperature dependence of the rate coefficients for reaction R1. Both the experiments and the QCT calculations cover a wide temperature range between 225 and 960 K. The new experimental points are used to check the validity of the switch of the sign of the activation energy predicted by the QCT calculations.

In the rest of the paper, we first describe the experimental and theoretical methodology followed by the presentation of the new experimental and theoretical rate coefficients and their comparison with the existing experimental results. Then, we discuss how the remarkable change as a function of temperature of the activation energy of reaction R1 can be reconciled with the existing thermochemical information.

## Methods

### Experiments

Kinetic measurements have been performed at a total pressure of 2 Torr of helium in a flow tube reactor combined with an electron impact ionization quadrupole mass spectrometer (operated at 30 eV energy) for the detection of the gas phase species. The experimental setup has been extensively used in the past to study the kinetics and products of the reactions involving a variety of atoms and radicals, in particular the reaction of HBr with OH and its isotopic analogues.^30,31^

Two different flow reactors were used, one for low-temperature measurements and one for high-temperature measurements.

A low-temperature flow reactor (used at 225–320 K) consisted of a Pyrex tube (45 cm in length, 2.4 cm i.d.) surrounded by a jacket through which thermostatted ethanol was circulated. The inner surface of the reactor was coated with halocarbon wax to reduce the wall-loss of active species (F atoms and CH_3_ radicals). A high-temperature flow reactor (Figure S1) was employed over the temperature range 300–960 K and consisted of a quartz tube (45 cm in length, 2.5 cm i.d.), where the temperature was controlled with electrical heating elements.^32^

CH_3_ radicals were produced in the movable injector (Figure S1) in reaction of F atoms with excess CH_4_ ([CH_4_] = (3–5) × 10^13^ molecule cm^–3^):^33^
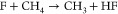
R2

*k*_2_ = 1.28 × 10^–10^ exp(−219/*T*) cm^3^ molecule^–1^ s^–1^ (*T* = 220–960).

Fluorine atoms were produced in a microwave discharge of trace amounts of F_2_ in He. It was verified by mass spectrometry that more than 95% of F_2_ was dissociated in the microwave discharge. CH_3_ radicals were detected either as CH_3_Br (CH_3_Br^+^, *m*/*z* = 94) or as CH_3_I at *m*/*z* = 142 (CH_3_I^+^) after being scavenged in rapid reactions with excess Br_2_ ([Br_2_] = (5–6) × 10^13^ molecule cm^–3^) or I_2_ ([I_2_] = (4–5) × 10^13^ molecule cm^–3^), respectively, added at the end of the reactor 5 cm upstream of the sampling cone (Figure S1):
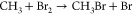
R3

*k*_3_ = 1.83 × 10^–11^ exp(252/*T*) cm^3^molecule^–1^ s^–1^ (*T* = 224–358 K).^34^
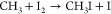
R4

The rate constant of reaction R4 is not well known but can be expected to be at least as high as that for reaction R3. In all cases, we observed a total conversion of CH_3_ to CH_3_I under the experimental conditions used. Absolute calibration of the mass spectrometric signals of CH_3_Br was carried out as follows. First, the F atoms were titrated with an excess of Br_2_ in the main reactor, which led to the formation of FBr ([FBr]_0_):
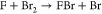
R5

*k*_5_ = (1.28 ± 0.2) × 10^–10^cm^3^molecule^–1^ s^–1^ (*T* = 299–940 K).^35^

Then, the same concentration of F atoms was titrated with a mixture of Br_2_ and CH_4_, resulting in the formation of FBr ([FBr]) and CH_3_ in reactions R5 and R1, respectively. In the presence of Br_2_, CH_3_ radicals are rapidly converted to CH_3_Br according to reaction R3. The absolute concentration of CH_3_Br was determined as [CH_3_Br] = [FBr]_0_ – [FBr]. This calibration procedure avoids possible complications due to the self-reaction and wall loss of CH_3_ radicals. Absolute concentrations of FBr were determined upon titration of F atoms in reaction R5 from the consumed fraction of Br_2_ ([FBr] = Δ[Br_2_]).

HBr vapor was delivered to the reactor from a flask with a known gaseous HBr/He mixture and was detected by mass spectrometry at its parent peak of *m*/*z* = 80 (HBr^+^). Mass spectrometric analysis showed that no noticeable decomposition of HBr occurred when storing HBr/He mixtures in a glass flask for weeks. The concentration of the potential decomposition product, Br_2_, was estimated to be less than 0.1% of that of HBr. The absolute calibration of the mass spectrometer for HBr was realized using two methods. The first one employed chemical conversion of a H-atom to HBr in reaction with excess Br_2_
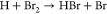
R6

In this way, the concentration of HBr formed in reaction R6 was related to the fraction of Br_2_ consumed. In the second method, the absolute concentration of HBr was calculated from the flow rate obtained from the measurements of the pressure drop of the manometrically prepared HBr/He mixture stored in a calibrated volume flask. The absolute concentrations of HBr determined with the two methods employed were consistent within a few percent. The absolute concentrations of the other stable species (CH_4_, Br_2_, and F_2_) in the reactor were derived from their flow rates. I_2_ was introduced into the reactor by flowing helium through a column containing iodine crystals. The absolute calibration of I_2_ was realized using a method linking concentrations of I_2_ and Br_2_. It consisted of a titration of the same concentration of F atoms with excess Br_2_ ([F]_0_ = Δ[Br_2_]) and I_2_ ([F]_0_ = Δ[I_2_]). This procedure allowed the absolute calibration of I_2_ signals using that of Br_2_.

The purities of the gases used were as follows: He >99.999% (Alphagaz); Br_2_ > 99.99% (Aldrich); I_2_, 99.999% (Aldrich); F_2_, 5% in helium (Alphagaz); HBr > 99.8% (Praxair); CH_4_ > 99.995% (Alphagaz).

### Quasiclassical Trajectory Simulations

The calculations were performed using the potential energy surface function developed by Czakó^23^ corrected as described in ref (28). The standard QCT technology was used,^36^ described in more detail in refs (27−38). The calculations were performed using an extensively modified version of the trajectory code VENUS 88.^39^ The Monte Carlo method was used to sample the internal energies of the reactants and the relative translational energy from the Boltzmann distribution. The impact parameter was sampled without weighting up to a maximum value, *b*_max_, which was determined in exploratory calculations, and varied from 4.5 Å at 200 K to 11.0 Å at 1000 K. 200,000 trajectories were run at every temperature. Energy conservation was better than 0.05 kJ/mol.

## Results

### Measurements of *k*_1_

**Figure 1:**
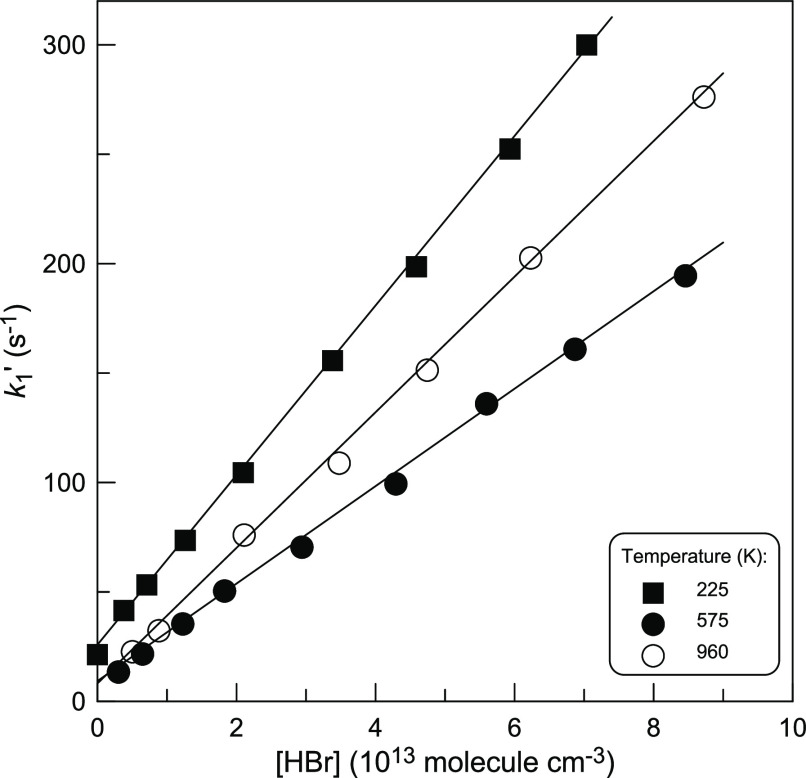
Pseudo-first-order rate constant (*k*_1_′) as a function of the concentration of HBr at different temperatures.

The measurements of *k*_1_ were carried out under pseudo-first-order conditions, monitoring the kinetics of CH_3_ consumption ([CH_3_]_0_ = (0.6–1.2) × 10^11^ molecule cm^–3^) in an excess of HBr (see Table 1 for the concentrations of HBr) by changing the position of the movable injector (Figure S1). The distance between the injector head and the Br_2_ (I_2_) introduction point (5 cm upstream of the sampling cone) was converted into reaction time using the linear flow velocity (1550–1940 cm s^–1^) of the gas mixture in the reactor. Figure S2 shows typical examples of the observed concentration *vs* time profiles of CH_3_ radicals. The decays of CH_3_ radicals are first order, [CH_3_] = [CH_3_]_0_ × exp(−*k*_1_′ × *t*), where *k*_1_′ = *k*_1_ × [HBr] + *k*_w_ is the pseudo-first-order rate constant with *k*_w_ representing the heterogeneous loss of CH_3_ radicals. Examples of the typical second-order plots observed at different temperatures are shown in Figure 1.

**Table 1:** Summary of the Present Measurements of *k*_1_

*T* (K)^a^	[HBr] (10^13^ molecule cm^–3^)	*k*_1_ (±2σ)^b^ (10^–12^ cm^3^ molecule^–1^ s^–1^)	reactor surface/CH_3_ detection^c^
225	0.38–7.04	3.85 ± 0.07	HW/CH_3_Br
235	0.55–9.45	3.66 ± 0.06	HW/CH_3_Br
250	0.83–10.7	3.47 ± 0.06	HW/CH_3_Br
270	0.64–9.93	3.20 ± 0.08	HW/CH_3_Br
295	0.88–13.2	3.02 ± 0.05	HW/CH_3_Br
300	0.48–5.27	3.05 ± 0.07	Q/CH_3_Br
320	0.74–11.3	2.85 ± 0.06	HW/CH_3_Br
360	0.74–6.35	2.67 ± 0.08	Q/CH_3_Br
380	0.96–13.1	2.56 ± 0.05	Q/CH_3_I
410	0.74–8.90	2.44 ± 0.08	Q/CH_3_Br
475	0.48–8.73	2.32 ± 0.07	Q/CH_3_I
575	0.30–8.46	2.23 ± 0.06	Q/CH_3_I
720	0.33–9.54	2.36 ± 0.06	Q/CH_3_I
790	0.26–12.6	2.44 ± 0.04	Q/CH_3_I
830	0.53–9.77	2.51 ± 0.04	Q/CH_3_I
880	0.31–9.65	2.75 ± 0.04	Q/CH_3_I
960	0.50–8.72	3.10 ± 0.05	Q/CH_3_I

a 7–11 kinetic runs at each temperature.

b Total estimated uncertainty on *k*_1_ is about 10%.

c HW: halocarbon wax; Q: uncoated quartz; see text for CH_3_ detection.

The slopes of the straight lines in Figure 1 provide the bimolecular rate constants at the respective temperatures. A summary of the experimental measurements of *k*_1_ is given in Table 1. The combined uncertainty on *k*_1_ was estimated to be about 10% by adding in quadrature statistical error (<3%) and those on the measurements of the absolute concentration of HBr (∼7%), flows (3%), pressure (2%), and temperature (1%).

**Figure 2:**
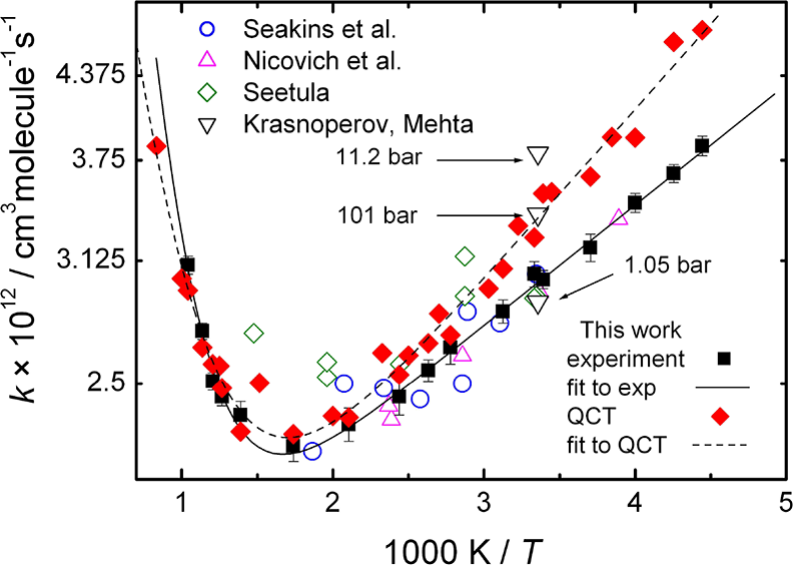
Measured (black squares) and calculated (red filled diamonds) rate coefficients for reaction CH_3_ + HBr → CH_4_ + Br (reaction R1). The lines correspond to fitted double-exponential functions whose parameters are listed in the text. The earlier experimental results are from Nicovich *et al.*(15) (magenta triangles), Seakins *et al.*(16) (blue circles), Seetula^17^ (green diamonds), and Krasnoperov and Mehta^40^ (black triangles). In ref (40), three measurements were performed at 1.05, 11.2, and 101 bar, as marked by the arrows. The pressure used in refs (15) and (16) was 0.013–0.4 bar, and in the present work, it is 0.0026 bar.

The present experimental data for *k*_1_ are plotted as a function of temperature in Figure 2 together with previous temperature-dependent measurements^15−17^ as well as with the QCT results.

Reaction R1 is fast, and the rate coefficients are between 2.2–4.5 × 10^–12^ cm^3^ molecule^–1^ s^–1^. The largest difference between the smallest and largest rate coefficients is only a factor of 2, and no pressure dependence can be observed. Spectacular is the strong tendency showing that the reaction of the CH_3_ radical with HBr clearly exhibits a non-Arrhenius behavior: negative temperature dependence is seen below 575 K, whereas positive temperature dependence is observed above 720 K. Thus, the experiments confirm that the activation energy is positive at high temperatures as expected based on the QCT and earlier TST-based predictions.

One can note the excellent agreement between the present data and those of Nicovich *et al*.^15^ and Seakins *et al*.^16^ The measurements of Seetula^17^ are also consistent with our data within the experimental uncertainties. The current experimental results have been fitted to a double exponential function (solid line in Figure 2):

*k*_1_(exp) = 1.44 × 10^–12^ exp(219/*T*) + 6.18 × 10^–11^ exp(−3730/*T*) cm^3^ molecule^–1^ s^–1^

This expression adequately describes all existing experimental data and is expected to be accurate within 15% between 225 and 960 K.

Remarkable is the agreement between all experimental results and the rate coefficients obtained by the QCT calculations, including the strong non-Arrhenius behavior. The optimal double-exponential fit to the QCT data is

*k*_1_(QCT) = 1.19 × 10^–12^ exp(309/*T*) + 1.89 × 10^–11^ exp(−2559/*T*) cm^3^ molecule^–1^ s^–1^

The location and magnitude of the minimum of the Arrhenius curve obtained experimentally and from trajectory simulations are essentially identical. Between 300 and 960 K, the data points obtained with the two methods differ only by a few percent. In the high-temperature wing, the rate of increase in both the experimental and QCT rate coefficients is very close. In the low-temperature wing, both the experiments and the QCT simulations yield negative activation energy, but in this wing, the QCT results are systematically larger than the experimental ones. The deviation is below the 10% estimated combined uncertainty of the experimental rate coefficients above about 300 K, but at lower temperatures, it exceeds it. The discrepancy of this magnitude can easily be the consequence of a slightly too strong long-range attraction between the reactants on the employed potential energy surface.

## Discussion

The experiments and the QCT calculations, which are in very good agreement, suggest that the activation energy of reaction R1 changes significantly with the temperature. The Arrhenius plot is highly curved, and it goes through a minimum and is close to linear at the limits of both the high and low temperatures. The activation energy, calculated as the negative of the local slope of the Arrhenius plot, is *E*_a_(*T* < 300 K, exp) = −1.82 kJ/mol according to the current experiments and *E*_a_(*T* < 300 K, QCT) = −2.57 kJ/mol from the QCT calculations. At the highest temperatures covered by the current measurements and calculations, above 800 K, the Arrhenius plot is virtually a straight line, but in fact, the activation energies calculated from the double-exponential fit to the experimental results are 6.4, 10.0, and 13.5 kJ/mol at 800, 900, and 1000 K, respectively. It is worth noting that the activation energy at 1000 K is 7.5 larger than the absolute value of *E*_a_ at the low-temperature limit.

The basic reaction kinetics information utilized in the determination of the heat of formation of the methyl radical according to the second-law method is the reaction enthalpy obtained as the difference of the activation energies:
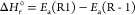
1

This relationship is valid under thermal equilibrium where the rates of the forward and reverse reactions are equal. In the derivation of the enthalpy of formation of the methyl radical, the activation energy for the reverse reaction, CH_4_ + Br → CH_3_ + HBr, was considered to be constant, 73.9 ± 2.5 kJ/mol,^14,16^ based on direct measurements of the rate of the (CH_3_)_3_CH + Br → (CH_3_)_3_C + HBr reaction and a series of relative rates determined earlier.^41,42^

**Figure 3:**
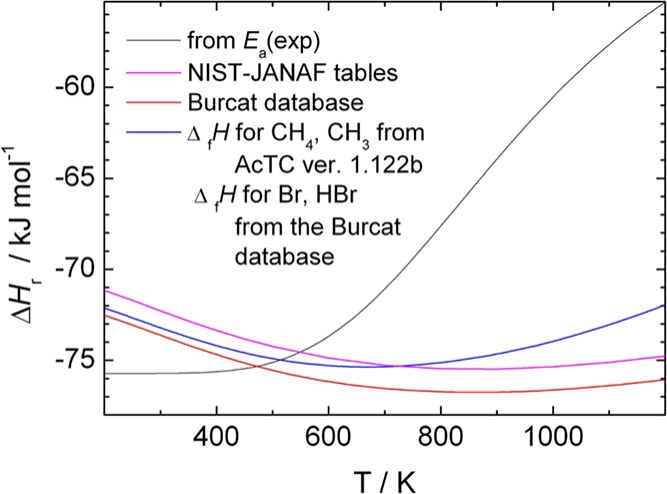
Comparison of the temperature dependence of the reaction enthalpy of reaction R1 calculated from the measured temperature-dependent forward and the constant *E*_a_(R-1) = 73.9 kJ/mol reverse activation energies and from the heats of formation of the reactants and products of R1 (taken from various databases^4,43,44^).

Considering that the activation energy for the methyl + HBr reaction changes by about 15 kJ/mol between 200 and 1000 K, the reaction enthalpy calculated from the activation energy determined in this work and from the literature value^14,16^ of *E*_a_(R-1) = 73.9 kJ/mol will vary significantly: from about −75.72 kJ/mol at temperatures below cca. 400 K to about −57.65 kJ/mol at 1000 K. The exothermicity calculated from the activation energies would decrease further above 1000 K. This significant change is hard to reconcile with the currently accepted thermochemical data. Figure 3 compares the reaction enthalpies calculated from the activation energies and from the tabulated heats of formation. One can see that the absolute value of the enthalpy of reaction R1 obtained from the activation energies increases by ∼20 kJ/mol between 200 and 1200 K, while from the tabulated heats of formation of CH_4_ and Br as well as CH_3_ and HBr, the change of Δ_r_*H*°(R1) is within a range narrower than 4.5 kJ/mol and is not monotonic.

A consequence of this deviation is that if one calculates the enthalpy of formation of CH_3_ from the reaction enthalpy obtained from the activation energies of R1 and R-1 as it was done in the earlier experiments,^14−16^ its value and its temperature dependence differ from what one derives from data in thermochemical tables. Using the enthalpies of formation of the three other species involved in the reaction taken from the 1.122b version of ATcT and the reaction enthalpy from the activation energies one obtains, Δ_f_*H*°(CH_3_, 298.15) = 148.9 kJ/mol instead of the 146.46 kJ/mol according the same version of ATcT. More serious deviation is that, compared with what one obtains using the NASA polynomials for CH_3_, the rate of increase of Δ*H*_f_^°^(CH_3_) with temperature is slightly too slow below 500 K, and much more too slow between 500 and 1000 K so that at 1000 K, the deviation from the tabulated value is as large as −16.5 kJ/mol.

The large difference appears above 500 K, which coincides with the change of sign of the activation energy. The inconsistency of the data obtained from the activation energies for the forward and reverse reaction (the former measured in the present work, the latter being the accepted constant value) can be resolved by assuming that either the measured positive activation energy at high temperatures is an artifact or that the activation energy of the reverse reaction is not constant. Now, we investigate these two options.

On the validity of the change of the activation energy of the forward reaction: It is known that the activation energy of reactions passing through submerged barriers generally does change sign.^26,45^ In the current case, the very good agreement between the experiments and the QCT calculations supports not only the change of sign but even the magnitude of the activation energy at high temperatures. In addition, as shown in ref (28), the rate coefficients calculated with the QCT method agree very well with the extended Arrhenius expression derived by evaluation of literature data in ref (46), which also yields positive activation energy above 500 K. Based on this, it is reasonable to assume that the temperature dependence of the rate coefficient for reaction R1 and of the activation energy is correct.

The other constituent of the reaction enthalpy, the activation energy of the reverse reaction, has not been determined in direct experiments; instead, it is based on *E*_a_ of the Br + i-butane reaction determined below 500 K and a series of relative rates.^14,16^ When the enthalpy of formation of the methyl radical was evaluated and the activation energy of reaction R1 measured at low temperatures was combined with the value *E*_a_(R-1) = 73.9 kJ/mol, the obtained value proved to be consistent with that derived from other sources. The combination of low-temperature activation energies looks reasonable, considering that they are very probably close to constant in that temperature range. The large deviation between the reaction enthalpies obtained from tabulated data and those derived via the combination of the *E*_a_(R1) determined in this work and the constant 73.9 kJ/mol for the reverse reaction suggests that at higher temperatures, the activation energy of the CH_4_ + Br reaction should also change. The magnitude of this change can be estimated by combining the *E*_a_(R1) measured in this work with the Δ_r_*H*°(R1) calculated from tabulated heats of formation. We consider the tabulated values together with their temperature dependence as solidly founded, in particular in light of the consistency provided by the active thermochemical tables. The temperature dependence reported in tabulations is based on statistical mechanical calculations of the partition function, which, although are approximate, provide results that also proved to be consistent within quite an extended thermochemical network. This suggests that the only parameter whose temperature dependence can deviate from the assumed constant value is the activation energy of the CH_4_ + Br reaction.

When one calculates this activation energy from the *E*_a_(R1) measured in this work and Δ_r_*H*°(R1) calculated from the tabulated heats of formation, one gets *E*_a_(R-1) = 69.2 kJ/mol in the limit of zero kelvin. This can be compared with the reaction enthalpy for the reverse reaction because, although there is a submerged potential barrier, the activation energy (in Tolman’s sense as “the average energy of the reacted reactants”) must cover the reaction endothermicity.

The Δ_r_*H*°(R-1) is 71.5 kJ/mol according to data from Burcat’s database, 70.0 kJ/mol is obtained from heats of formation in ATcT ver. 1.122b, and a value of 70.2 kJ/mol was derived from highly accurate ab initio calculations by Czakó.^23^ The observation that the “reverse-engineered” zero-kelvin activation energy agrees very well with the reaction enthalpy derived from various sources suggests that the energy available for the reactants is fully utilized for climbing from the CH_4_ + Br to the CH_3_ + HBr energy level. In the temperature range where the experiments for the reaction of i-butane and Br were performed, one obtains for *E*_a_(R-1) the values 71.2 and 72.5 kJ/mol at 298.15 and 400 K, respectively. These values are close to the 73.9 kJ/mol derived from rate measurements of other reactions. Furthermore, the Δ_r_*H*°(R_1_) calculated from the measured *E*_a_(R_1_) and *E*_a_(R-1) = 73.9 kJ/mol is reasonably close to that taken from the thermochemical databases. This explains the success of the earlier experiments in determining the correct enthalpy of formation for CH_3_ using *E*_a_(R-1) = 73.9 kJ/mol. Different is the situation at higher temperatures.

**Figure 4:**
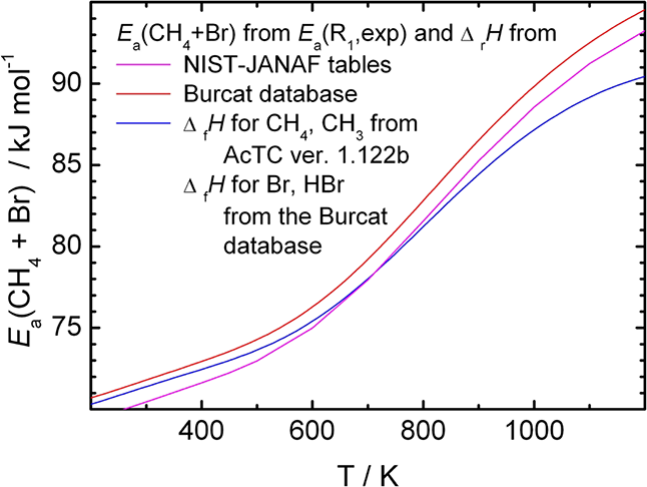
Temperature dependence of *E*_a_(R-1) calculated from the activation energy for the forward reaction and the reaction enthalpy from the heats of formation taken from databases.

The temperature dependence of *E*_a_(R-1) derived for the markedly temperature-dependent of *E*_a_(R1) measured in this work and the much less temperature-dependent Δ_r_*H*°(R_1_) derived from thermochemical tables is shown in Figure 4 to be as large as that of *E*_a_(R_1_). While the change of the latter with the temperature can be explained by the presence of the pre-reaction complex and the submerged barrier, the reason why the activation energy of a significantly endothermic reaction should increase above the reaction enthalpy as much as we found is not obvious. In terms of transition state theory, the transition states, the “tight” one corresponding to the potential barrier, and the “loose” corresponding to the centrifugal barrier between the van der Waals well and the separated CH_3_ radical and HBr molecule reactants govern the rate of both the forward and the reverse reaction. However, the lifetime distribution of the reactive trajectories in the van der Waals well^27^ as well as the weak communication between the intra- and interfragment modes suggests that the energy redistribution is probably not very fast, *i.e.*, there is not necessarily equilibrium between the modes. This means that one of the conditions of applicability of the RRKM theory (the version of transition state theory apt for such systems) is not fulfilled. The earlier QCT calculations^27^ on the CH_3_ + HBr reaction also indicated that a significant fraction of trajectories, at low energies up to around 50%, recross the region of the submerged potential barrier, which undermines the other basic assumption of transition state theory.

It is worth mentioning that in the experimental studies of the higher alkane analogs^13,21^ of the CH_4_ + Br reaction, there is no sign of temperature dependence of the activation energy; note, however, that the temperature in those studies was well within the essentially linear high-temperature region of the presumedly curved Arrhenius plot.

A possible way of understanding the change of activation energy of reaction R-1 is dynamical simulations. QCT calculations on the CH_4_ + Br reaction are in progress in our laboratories.

## Conclusions

The rate coefficients obtained in the current experiments for the CH_3_ + HBr reaction agree very well with the literature data available at temperatures below about 500 K. The activation energy in this region is negative. The QCT calculations also reproduce the experimental rate coefficients in this region and support the negative sign of the activation energy. At high temperatures, however, according to both the experiments and the simulations, the activation energy is positive and the slope of the Arrhenius plot is larger than at low temperatures. This behavior was already seen in our earlier QCT calculations and confirmed in the current ones.

The magnitude of the activation energy changes significantly, by about 15 kJ/mol between 225 and 1000 K. The reaction enthalpy calculated from the tabulated heats of formation of the four species involved in the reaction changes by at most a factor of three less than the activation energy measured/calculated for the CH_3_ + HBr reaction. To reconcile the two observations, one must assume that the activation energy of the reverse CH_4_ + Br reaction changes parallel to that of the forward reaction.

## Supporting Information Available

(Figure S1) Diagram of the high-temperature flow apparatus and (Figure S2) typical pseudo-first-order plots (PDF)
