# Supplementary material for: Experimental and Theoretical Study of the Kinetics of the CH3 + HBr → CH4 + Br Reaction and the Temperature Dependence of the Activation Energy of CH4 + Br → CH3 + HBr
Source: J Phys Chem A. 2023 Aug 10;127(33):6916–23. doi: 10.1021/acs.jpca.3c03685 (PMC10461296; doi:10.1021/acs.jpca.3c03685)
Supplement: Supplementary file 1 — jp3c03685_si_001.pdf [file jp3c03685_si_001.pdf]

Supporting Information
Manuscript No. jp-2023-03685y

Experimental and Theoretical Study of the Kinetics of the CH_3 + HBr → CH_4 + Br Reaction and the Temperature Dependence of the Activation Energy of CH_4 + Br → CH_3 + HBr

Yuri Bedjanian[*1], Péter Szabó[2,3] and György Lendvay*[*4,5]
[1] Institut de Combustion, Aérothermique, Réactivité et Environnement (ICARE), CNRS
45071 Orléans Cedex 2, France
[2] Department of Chemistry, KU Leuven, Celestijnenlaan, 200F
3001 Leuven, Belgium
[3] Royal Belgian Institute for Space Aeronomy (BIRA-IASB),
Avenue Circulaire 3, 1180 Brussels, Belgium
[4] Institute of Materials and Environmental Chemistry, Research Centre for Natural Sciences,
Magyar tudósok krt. 2., H-1117 Budapest, Hungary
[5] Center for Natural Sciences, Faculty of Engineering, University of Pannonia, Egyetem u. 10.
Veszprém, 8200 Hungary

Contents

Figure S1. Diagram of the high temperature flow reactor: configuration used in the measurements of k_1.
Figure S2. Typical CH_3 decay profiles in the presence of different concentrations of HBr measured at T = 320 K.

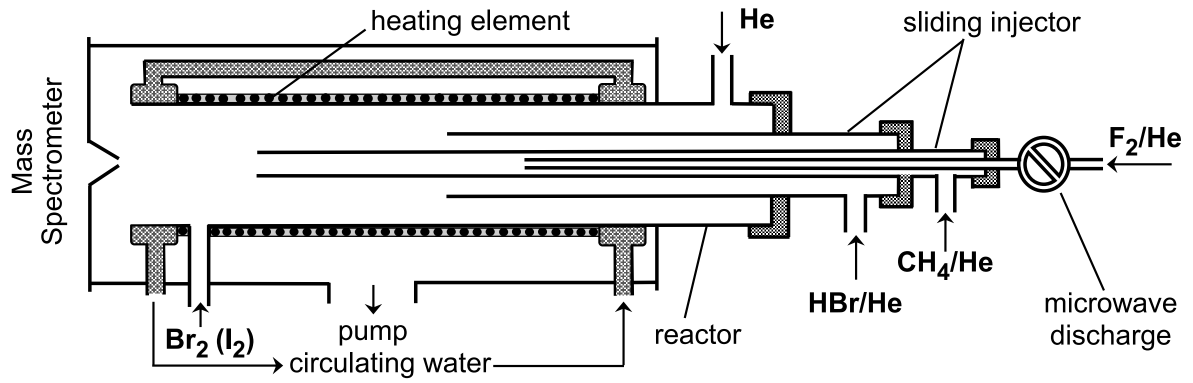

Figure S1. Diagram of the high temperature flow reactor: configuration used in the measurements of k_1.

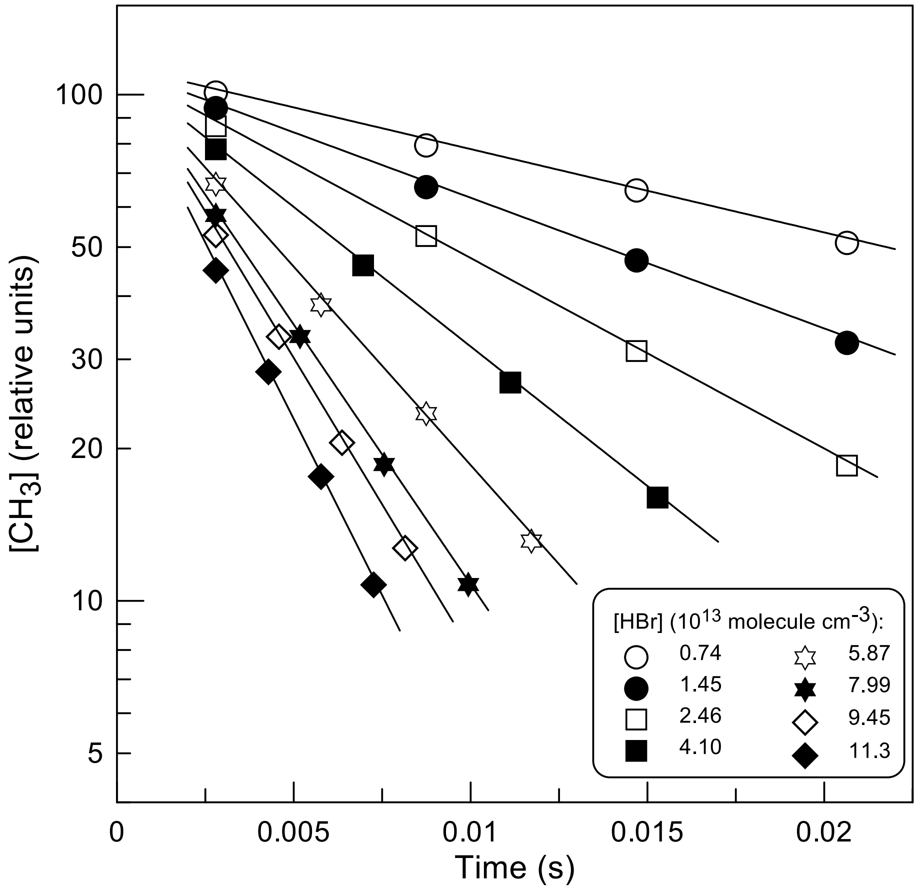

Figure S2. Typical CH_3 decay profiles in the presence of different concentrations of HBr measured at T = 320 K.
